# Analysis of current status and potential categories of nutritional literacy in elderly patients with chronic diseases: a single-center cross-sectional study

**DOI:** 10.3389/fnut.2025.1670339

**Published:** 2025-10-02

**Authors:** Zhitong Wang, Wenxiu Jiang, Jialin Wang

**Affiliations:** ^1^School of Nursing, Chengdu University of Traditional Chinese Medicine, Chengdu, China; ^2^Department of Geriatrics, Deyang People’s Hospital, Deyang, China; ^3^Department of Pediatrics, First Affiliated Hospital of Chengdu Medical College, Chengdu, China

**Keywords:** elderly, patients with chronic diseases, nutrition literacy, chronic disease self-efficacy, latent class analysis

## Abstract

**Objective:**

This study identifies nutritional literacy subgroups and influencing factors among elderly chronic disease patients to develop targeted interventions.

**Methods:**

A total of 316 elderly chronic disease patients from a tertiary hospital in Deyang, China, were surveyed using standardized scales (General Information, Nutritional Literacy, Chronic Disease Management Self-Efficacy). Latent profile analysis identified nutritional literacy subgroups, with univariate and ordered logistic regression verifying influencing factors.

**Results:**

A total of 316 valid samples were included, with a nutritional literacy score of 67.50 (51.00, 84.00). Nutritional literacy among elderly patients with chronic diseases was classified into three latent categories: “passive dependent type” (23.1%), “cognitive fluctuation type” (42.4%), and “autonomous management type” (34.5%). Ordered logistic regression showed that younger age groups had 25.64-fold [95% confidence interval (CI):5.07–129.66], 13.93-fold (95% CI: 3.10–62.61), and 9.66-fold (95% CI:2.42–38.54) higher odds of better nutritional literacy compared to the reference group aged ≥90 years. Compared to the highest income group (≥5,001 yuan), lower monthly income levels were associated with reduced odds of better nutritional literacy, with odds of 0.13-fold (95% CI: 0.04–0.45), 0.31-fold (95% CI: 0.11–0.91), and 0.25 -fold (95% CI: 0.09–0.64) for the ≤1,000 yuan, 1,001–3,000 yuan, and 3,001–5,000 yuan brackets, respectively. Smartphone use was associated with 1.96-fold higher odds versus non-users (95% CI: 1.03–3.74), while each unit increase in self-efficacy for chronic disease management was associated with 1.04-fold higher odds (95% CI:1.01–1.07) (all *p* < 0.05).

**Conclusion:**

Nutritional literacy among elderly patients with chronic diseases is moderately low and heterogeneous, and can be categorized into three latent profiles. Different categories of elderly patients exhibit distinct nutritional characteristics; therefore, interventions should be tailored to each nutritional literacy category to develop personalized improvement strategies. In particular, more education and support should be provided to “passive dependent type” patients to help enhance their self-management ability and improve their nutritional status, thereby better managing their chronic diseases and improving their quality of life. However, this single-center convenience sample from one Chinese city limits generalizability due to unaccounted regional/cultural diversity. Future multi-center studies across diverse settings should validate these findings.

## 1 Introduction

According to the report of the National Bureau of Statistics, by the end of 2023, the proportion of China’s population aged 60 years or older reached 18.1%. Among them, 15.4% were aged 65 years or older (1). The 14th Five-Year Plan for Healthy Aging states that 78% of the elderly population in China suffer from chronic diseases. It is expected that by 2030, the proportion of the elderly population will be close to 27%. Consequently, the number of people suffering from chronic diseases will triple, and the burden of chronic diseases will increase by at least 40% (2, 3). Chronic diseases have become the main cause of death for Chinese residents, accounting for 88.5% of all deaths (4). China is facing the dual challenges of an aging population and an increasing burden of chronic diseases. Protecting the health of the growing number of older adults with chronic diseases presents a major test for the high-quality development of medical and health services. The National Nutrition Program (2017–2030) clearly states that nutrition plays a crucial role in the prevention and management of certain chronic diseases, and actively promoting nutritional interventions can effectively improve patients’ health outcomes (5). Existing studies (6, 7) have shown that malnutrition (including undernutrition and overnutrition) is closely associated with a high incidence of chronic non-communicable diseases. Moreover, the vulnerability of older adults to malnutrition, combined with declining organ and immune functions, contributes to the high prevalence of chronic diseases in this population. Therefore, to improve the health outcomes of older adults with chronic diseases, it is necessary to enhance nutritional management strategies for this population.

In recent years, the nutritional status of the elderly has improved as the living standards of the Chinese people continue to rise; however, nutritional problems still exist. According to the monitoring report on the nutrition and health status of the Chinese population (8), the dietary intake structure of most elderly individuals in China is irrational, leading to the coexistence of undernutrition and overnutrition. These problems are closely related to lower levels of nutritional literacy, overall poorer nutritional knowledge, attitudes, and behaviors, as well as the prevalence of poor dietary habits among the elderly (9, 10). Nutritional literacy refers to an individual’s ability to obtain and understand nutritional information and make sound nutritional decisions (11). A study by Taylor et al. (12) found that the level of nutritional literacy predicted compliance with healthy and unhealthy dietary patterns, and that poorer nutritional literacy may reduce an individual’s ability to make healthy dietary choices, while good nutritional literacy promotes such choices. In recent years, compliance with nutritional recommendations and self-efficacy of patients with chronic diseases have been improved through nutritional literacy interventions. Moreover, the incidence of related complications has been reduced, leading to decreased healthcare expenditures (13). It is thus clear that, in order to improve the clinical outcomes of elderly patients with chronic diseases, nutritional literacy interventions should be carried out to enhance the nutritional literacy level of this population. However, current research on nutrition in the elderly mainly focuses on risk management and intervention, whereas there are fewer studies on nutritional literacy in the elderly population, especially among elderly patients with chronic diseases. Most of these studies are confined to single-center surveys and analyses of the current situation, which hinders the development of intervention studies (14). In-depth exploration of multidimensional influencing factors is urgently needed to improve the level of nutritional literacy in this group.

Social Cognitive Theory (SCT) originated in the mid-20th century and was proposed by the renowned psychologist Albert Bandura. It aims to explain the theoretical framework of how individuals learn and behave within their environments (15). SCT covers three aspects: an individual’s cognition, the environment, and personal behavior. It focuses on an individual’s motivations to perform health behaviors and translates these motivations into behavioral intentions to effectively predict health behaviors (15). In the field of health promotion, SCT is widely used to design and evaluate measures to improve health literacy (16–19). As a specific form of health literacy, nutritional literacy fits within the scope of application of the social cognitive theory. Moreover, social cognitive theory emphasizes the necessity of integrating environmental and individual cognitive factors, as well as the importance of highlighting self-efficacy within individual cognition. This integrated approach provides a comprehensive framework for understanding individual behaviors and lays the theoretical foundation for individuals to achieve self-regulation of healthy habits (20). Based on these principles, this study used social cognitive theory as a framework to incorporate chronic disease management self-efficacy into the current status survey and profile analysis of nutritional literacy in elderly patients with chronic disease. The study aimed to explore the potential categories of nutritional literacy and their influencing factors in this population to ensure that the results are scientific and comprehensive. Additionally, it aims to provide data support for the future development of interventions targeting nutritional literacy in elderly patients with chronic disease. The ultimate goal is to improve their nutritional health status through these.

## 2 Objects and methods

### 2.1 Subjects

Convenience sampling method was used to select elderly patients with chronic diseases in a tertiary hospital in Deyang City, Sichuan Province, China, from January to June 2025 as the study subjects. Inclusion criteria: (1) age ≥ 60 years; (2) no speech or hearing dysfunction; (3) Patients met the types of chronic diseases specified in the International Statistical Classification of Diseases and Related Health Problems, Tenth Revision (2nd Edition) (ICD-10) (21); (4) informed consent and voluntary participation in this study. Exclusion criteria: (1) Acute illness or cognitive impairment that prevented them from cooperating with the survey; (2) Concurrent participation in other studies. The sample size calculation for this study was primarily based on the methodological requirements for latent profile analysis (LPA). According to established LPA methodology (22), the sample size must meet two minimum requirements: (1) at least 50 cases per continuous variable used in the model, and (2) at least 50 cases per anticipated latent class. For this study, the LPA model incorporated three nutritional literacy dimensions and two chronic disease self-efficacy dimensions, totaling five continuous variables. This resulted in a minimum requirement of 5 × 50 = 250 cases. Considering an anticipated 3–5 latent classes, with a maximum of 5 classes × 50 cases = 250 cases, the baseline sample size requirement remained 250 cases. To account for potential invalid responses or dropout, estimated at 20% based on prior studies, the target sample size was calculated by dividing the minimum sample size by (1—dropout rate): 250 ÷ (1–0.2) = 312.5, rounded upto 313 cases. This sample size would ensure stable parameter estimation and allow for model fit validation such as achieving entropy greater than 0.8, which indicates good classification quality in LPA. This study was approved by the Ethics Committee of the People’s Hospital of Deyang City, Sichuan Province (2022–04-010-K01).

### 2.2 Methodology

#### 2.2.1 Survey instruments

##### 2.2.1.1 General information questionnaire

The general information questionnaire, designed by our research team, includes 17 items covering demographic, socioeconomic, health-related, and behavioral variables. These items are: gender; age; marital status; body mass index (BMI); serum albumin levels; number of children; education level; place of residence; primary caregiver; current and pre-retirement occupation; personal monthly income; type of health insurance; type(s) of chronic diseases; use of multiple medications; frequency of watching television; frequency of reading books; use of smartphones; and oral health status.

##### 2.2.1.2 Nutritional literacy scale for the elderly

The Nutritional Literacy Scale for the Elderly was developed by Fang (23) and is widely used in the elderly population. The scale consists of 3 dimensions, namely functional nutritional literacy, interactive nutritional literacy, and critical nutritional literacy, with a total of 23 items. The scale is rated on a 5-point Likert scale. The response options are: Strongly Disagree = 1, Disagree = 2, Neutral = 3, Agree = 4, and Strongly Agree = 5. The total score of the scale ranges from 23 to 115, with higher scores indicating higher levels of nutritional literacy among the elderly. In this study, the Cronbach’s alpha coefficient of the scale was 0.960. The total scale item-level content validity index (I-CVI) ranged from 0.83 to 1.00, and the scale-level content validity index (S-CVI) was 0.90.

##### 2.2.1.3 Chronic disease self-efficacy scale

The Chronic Disease Self-Efficacy Scale (CDSES) was developed by Lorig and Holman (24) at Stanford University in the USA. The Chinese version was developed by Siu et al. (25). The scale consists of six items in two dimensions: Symptom Management Self-Efficacy (four items) and Disease Co-Management Self-Efficacy (two items). Specifically, the scale is rated on a 10-point Likert scale, with 1 indicating “not at all confident” and 10 indicating “completely confident.” The total scale score ranges from 6 to 60. The evaluation criterion is the mean item score, with ≤4.0 as low level, 4.0–7.9 as medium level, and ≥8.0 as high level. Cronbach’s alpha of the scale ranges from 0.88–0.95. In this study, Cronbach’s alpha is 0.976. The item-level content validity index (I-CVI) of the scale ranges from 0.92 to 1.00, and the scale-level content validity index (S-CVI) is 0.97.

#### 2.2.2 Survey methods

This study was conducted using a face-to-face questionnaire. First, the research team edited the content of the questionnaire, including the informed consent information, survey background, purpose, content, significance, instructions for completion, and confidentiality. The final version was determined after refining it according to the results of the pre-survey. Second, after obtaining informed consent, the two trained investigators assisted the subjects of the study in completing the questionnaire. For illiterate subjects, the investigators provided assistance after the subjects were fully informed. Finally, all completed questionnaires were collected anonymously, and the data were coded to ensure confidentiality and viewed only by members of the project team.

#### 2.2.3 Statistical analysis

Mplus 8.3 software was used to conduct latent profile analysis (LPA) for nutritional literacy, and model fit was assessed by increasing the number of profiles stepwise. The fit indices included the Akaike Information Criterion (AIC), Bayesian Information Criterion (BIC), and adjusted BIC (aBIC), with smaller values indicating better model fit. Information entropy (0–1 range) evaluated classification accuracy, where values closer to 1 indicated higher accuracy. The Lo–Mendell–Rubin adjusted likelihood ratio test (LMR-LRT) and Bootstrap Likelihood Ratio Test (BLRT) were employed, with significant *p*-values indicating that the k-class model fit better than the (k-1)-class model. While these indices guided model selection, the interpretability of profiles was also considered. The derived latent classes demonstrated a conceptual ordering reflecting progressive nutritional literacy levels (e.g., from basic to advanced), validated through threshold analysis showing clinically meaningful score differences between adjacent classes. For robustness verification, both ordered and multinomial models were run in parallel. The ordered multivariate logistic regression treated LPA classes as ordinal, while the multinomial model treated them as nominal categories. Results showed 88% concordance in predictor significance between models, with key differences observed in how self-efficacy variables predicted class transitions—the ordinal model captured overall progression patterns whereas the multinomial model identified specific between-class differences.

Data were statistically analyzed using SPSS 26.0 software. Continuous variables following a normal distribution were expressed as mean ± standard deviation, while those not normally distributed were expressed as median (interquartile range). Between-group comparisons for normally distributed continuous variables were made using analysis of variance (ANOVA). Categorical variables were expressed as frequencies and percentages. Between-group comparisons for categorical variables were made using the chi-square test, and for non-normally distributed continuous variables using the Mann–Whitney U test. Ordered multivariate logistic regression analyses were performed with the classification results of latent profile analysis (LPA) as the dependent variable, and the statistically significant variables in the univariate analyses as the independent variables. The significance threshold for type I error (*α*) was set at 0.05.

## 3 Results

### 3.1 General information of the respondents

A total of 330 questionnaires were collected this time, 14 questionnaires were filtered out due to inconsistent options or obvious logical errors, all of which were excluded, and the final confirmed valid questionnaires were 316, with an effective recovery rate of 95.8%. The age of elderly patients with chronic diseases ranged from 60 to 96 years. Based on established epidemiological categorization for older adult populations and clinical relevance to geriatric nutritional status, age was classified into four groups: 53 patients aged 60–64 years (16.8%), 103 patients aged 65–74 years (32.6%), 145 patients aged 75–89 years (45.9%), and 15 patients aged 90 years and above (4.7%). Among the respondents, there were 176 males (55.7%) and 140 females (44.3%). Regarding the chronic disease burden, 82 patients (25.9%) had one type of chronic disease, 181 patients (57.3%) had two to four types, and 53 patients (16.8%) had five or more types, with categorization reflecting distinct levels of clinical complexity. The monthly personal income (RMB) of respondents ranged from ≤1,000 yuan to ≥5,000 yuan. Income was categorized into four groups according to quartile distribution and socioeconomic stratification relevant to nutritional access in aging populations: 109 patients earning ≤1,000 yuan (34.5%), 82 patients earning 1,001–3,000 yuan (25.9%), 56 patients earning 3,001–5,000 yuan (17.7%), and 69 patients earning ≥5,001 yuan (21.8%). The linearity assumption in the logit was assessed using restricted cubic splines with three knots, which indicated non-linear relationships for both age and income, supporting the use of categorical terms for better model fit and clinical interpretability.

### 3.2 Nutritional literacy scale for elderly patients with chronic diseases and chronic disease self-efficacy scale scores of elderly patients with chronic diseases

Data in this study that did not conform to a normal distribution were expressed as median (interquartile range). The total score of the Nutritional Literacy Scale for Elderly Patients with Chronic Diseases was 67.50 (51.00, 84.00), indicating a score in the lower quartile of the moderate range. The score of the Chronic Disease Self-Efficacy Scale was 47.00 (38.00, 52.00), corresponding to the median of the upper moderate range. Detailed scores for each dimension are presented in Table 1.

**Table 1:** Scores of nutritional literacy scale and chronic disease self-efficacy scale in elderly patients with chronic diseases.

Scale	Dimension	Score [M (P25, P75)]	Reference value (score)
Nutritional literacy scale for the elderly	Functional nutrition literacy	32.00 (24.00, 39.00)	11 ~ 55
	Interactive nutrition literacy	18.00 (13.00, 23.00)	6 ~ 30
	Critical nutrition literacy	19.00 (15.00, 24.00)	6 ~ 30
	Total nutrition literacy score	67.50 (51.00, 84.00)	23 ~ 115
Chronic disease self-efficacy scale	Symptom management self-efficacy	31.00 (25.00, 34.75)	4–40
	Disease comorbidity management self-efficacy	16.00 (13.00, 18.00)	2–20
	Total self-efficacy score	47.00 (38.00, 52.00)	6–60

### 3.3 Characteristics and nomenclature of the latent profiles analysis of nutritional literacy in elderly patients with chronic diseases

Models with 1–5 categories were fitted using the 23-item scores of the Nutrition Scale as observed indicators (items) for LPA. Based on the model fit indices presented in Table 2 for latent profile analysis of nutritional literacy, the analysis of 1–5-class models using the 23-item Nutrition Scale demonstrated the following statistically supported pattern: Information criteria showed progressive improvement with increasing classes (AIC: 23,918.162 → 18,544.897; BIC: 24,090.926 → 19,078.212; aBIC: 23,945.026 → 18,627.824), though the magnitude of reduction substantially decreased beyond the 3-class solution (e.g., AIC reduction from 3-class to 4-class: 621.365 vs. 4-class to 5-class: 289.947). Entropy values consistently exceeded 0.90 (range: 0.962–0.973), confirming high classification accuracy across models. Critically, the Lo–Mendell–Rubin Adjusted LRT indicated statistically significant improvement for the 2-class (*p* < 0.001) and 3-class (*p* = 0.0003) models relative to their preceding models, while improvement was non-significant for the 4-class solution (*p* = 0.1106), establishing the 3-class model as the point of optimal statistical parsimony. The 3-class solution also demonstrated well-defined proportional distribution (23.1%/42.4%/34.5%), reinforcing clinical interpretability. These convergent statistical criteria—the attenuated improvement in information criteria beyond 3 classes, significant LMRT thresholds, and excellent classification quality (entropy = 0.973)—collectively validate the 3-class model as the optimal representation of nutrition literacy heterogeneity.

**Table 2:** Model fit indices of latent profile analysis for nutritional literacy in elderly patients with chronic diseases.

Category model	AIC	BIC	aBIC	Entropy	LMRT	BLRT (*p*-value)	Proportion of categories (%)
1	23918.162	24090.926	23945.026				
2	20655.768	20918.670	20696.648	0.970	0.0000	0.0000	50.9%/49.1%
3	19456.209	19809.249	19511.105	0.973	0.0003	0.0000	23.1%/42.4%/34.5%
4	18834.844	19278.021	18903.755	0.968	0.1106	0.0000	19.30%/31.96%/33.23%/15.51%
5	18544.897	19078.212	18627.824	0.962	0.3462	0.0000	17.41%29.43%/20.25%/12.66%/20.25%

**Figure 1:**
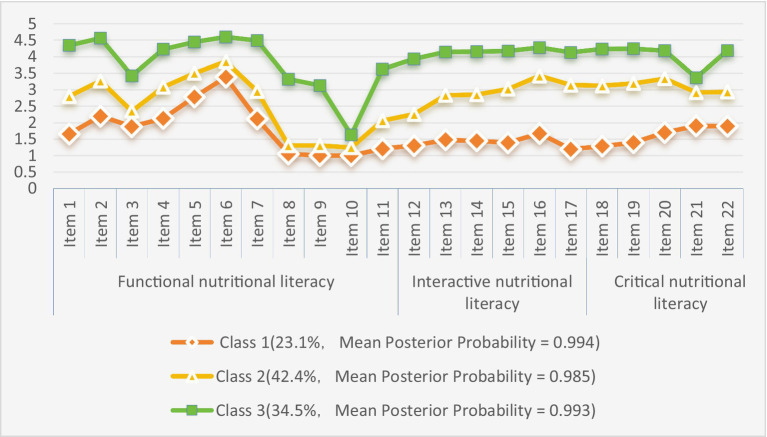
Distribution patterns of three latent profiles of nutritional literacy profiles in elderly patients with chronic diseases.

The potential profiles were plotted according to the classification results, as shown in Figure 1. The three profiles of nutritional literacy in elderly patients with chronic diseases did not intersect on any dimension, and their profile trends were relatively consistent, and each potential profile category was named according to its score on each entry.

Class 1 included 73 patients (23.1%), the smallest proportion. The patients in this category had low levels in the three dimensions of nutritional literacy: functional (e.g., application of basic knowledge), critical (e.g., analysis of information), and interactive (e.g., decision-making about health). They were weak in all aspects of nutritional literacy, which manifested as passive acceptance of external guidance and reliance on others to complete nutritional behaviors; therefore, this category was named “passive dependent type.”

Class 2 comprised 134 patients (42.4%), with scores at a medium level across all three dimensions of nutritional literacy. This indicates that patients in this category had some basic cognitive and health management abilities but were unstable in practice—sometimes rationally following scientific advice, sometimes easily influenced by the environment. This instability was reflected in the inconsistent knowledge base and the ability to apply it; thus, this category was named “cognitive fluctuation type.”

Class 3 included 109 patients (34.5%), whose scores in all three dimensions were at the highest level. Patients in this category actively integrated the three dimensions of literacy and continuously optimized their health management decisions through critical screening of information and systematic formulation of dietary strategies. Therefore, this category was named “autonomous management type.”

### 3.4 Univariate analysis of nutritional literacy potential profiles and population characteristics in elderly patients with chronic diseases

The results of univariate analysis showed that the distribution of nutrient literacy levels among elderly patients with chronic diseases differed significantly with respect to age, serum albumin levels, urban versus rural residence, marital status, number of children, Educational Attainment, monthly personal income, occupation, type of Medical Insurance and type of caregiver. Additionally, significant differences were observed according to Frequency of Reading, oral health status, Use of smartphone and level of self-efficacy in chronic disease management (*p* < 0.05), as shown in Table 3.

**Table 3:** Univariate analysis of three potential nutritional literacy profiles in elderly patients with chronic disease^a^.

Item	Passive dependent type	Cognitive fluctuation type	autonomous management type	Statistical value	*P*
Gender					
Male	37 (50.7%)	81 (60.4%)	58 (53.2%)	2.242^a^	0.326
Female	36 (49.3%)	53 (39.6%)	51 (46.8%)		
Age					
60–64 years	2(2.7%)	16(11.9%)	35(32.1%)	34.964^b^	<0.001
65–74 years	21(28.8%)	45(33.6%)	37(33.9%)		
75–89 years	41(56.2%)	69(51.5%)	35(32.1%)		
90 years and above	9(12.3%)	4(3.0%)	2(1.8%)		
Albumin (g/L)					
<30	2 (2.7%)	5 (3.7%)	2 (1.8%)	7.547^b^	0.023
30 to <35	7 (9.6%)	12 (9.0%)	4 (3.7%)		
−35 to <40	20 (27.4%)	34 (25.4%)	20 (18.3%)		
≥40	44 (60.3%)	83 (61.9%)	83(76.1%)		
Current address					
Countryside	52 (71.25%)	63(47%)	9 (8.3%)	78.622^a^	<0.001
Cities and towns	21 (28.8%)	71 (53%)	100 (91.7%)		
Marital status					
Married	48 (65.8%)	102 (76.1%)	96 (88.1%)	13.033^a^	0.001
Widowed or divorced	25 (34.2%)	32 (23.9%)	13 (11.3%)		
Educational attainment					
Primary school and below	66 (90.4%)	88 (65.7%)	23 (21.1%)	101.638 ^b^	<0.001
Junior high school	4 (5.5%)	31 (23.1%)	32 (29.4%)		
High school and above	3 (4.1%)	15(11.2%)	54(49.5%)		
Monthly personal income (RMB)					
≤1,000	52 (71.2%)	53 (39.6%)	4 (3.7%)	122.790^b^	<0.001
1,001–3,000	15 (20.5%)	42 (31.3%)	25 (22.9%)		
3,001–5,000	5 (6.8%)	26 (19.4%)	25 (22.9%)		
≥5,001	1 (1.4%)	13 (9.7%)	55 (50.5%)		
Number of children					
≤ 1	19 (26.0%)	36(30.5%)	63(53.4%)	32.455^b^	<0.001
2	11 (15.1%)	57 (42.5%)	24 (22.0%)		
3 and above	43 (40.6%)	41 (30.6%)	22 (20.2%)		
Occupation					
Physical labor	63 (86.3%)	97 (72.4%)	22 (20.2%)	101.700^a^	<0.001
Mental labor	6 (8.2%)	29 (21.6%)	74 (67.9%)		
Physical and mental labor	4 (5.5%)	8 (6.0%)	13 (11.9%)		
Type of caregiver					
Spouse	26 (35.6%)	64 (47.8%)	64 (58.7%)	15.597^a^	0.016
Children	19 (26%)	28 (20.9%)	10 (9.2%)		
Spouse and children	19 (26%)	32 (23.9%)	29 (26.6%)		
Others	9 (12.3%)	10 (7.5%)	6 (5.5%)		
Type of medical insurance					
Employee medical insurance	12 (16.4%)	56 (41.8%)	93 (85.3%)	90.816^a^	<0.001
New rural cooperative medical insurance	61 (83.6%)	78 (58.2%)	16 (14.7%)		
Number of chronic diseases					
1	27 (37%)	39 (29.1%)	16 (14.7%)	3.865^b^	0.145
2–4	29 (39.7%)	75 (56%)	77 (70.6%)		
≥5	17 (23.3%)	20 (14.9%)	16 (14.7%)		
Multiple medications					
≤1type	18 (24.7%)	36(26.9%)	24(30.8%)	0.336^b^	0.846
2–4 types	27 (37%)	50 (37.3%)	45 (36.9%)		
≥5types	28 (38.4%)	48 (35.8%)	40 (36.7%)		
Frequency of watching TV (hours/day)					
0	3 (4.1%)	19 (14.2%)	15 (13.8%)	4.471^b^	0.107
1–3	56 (76.7%)	91 (67.9%)	81 (74.3%)		
≥4	14 (19.2%)	24(17.9%)	13(11.9%)		
Frequency of reading (hours/day)					
0	70 (95.9%)	126 (94%)	81 (74.3%)	27.487^b^	<0.001
1–2	3 (4.1%)	7 (5.2%)	25 (22.9%)		
≥3	0	1 (0.7%)	3 (2.8%)		
Oral health use of smartphone					
Diseased	60 (82.2%)	113 (84.3%)	73 (67. 0%)	11.538^a^	0.003
Healthy	13 (17.8%)	21 (15.7%)	36 (33.0%)		
Yes	20 (27.4%)	78 (58.2%)	98 (89.9%)	73.970^a^	<0.001
No	53 (72.6%)	56 (41.8%)	11 (10.1%)		
CDSES (score)	42.0 (33.0, 48.0)	46.0 (37.0, 49.0)	51.0 (43.0, 54.5)	43.541^b^	<0.001

Statistical methods, ^a^χ^2^ test (Chi-square test); ^b^Kruskal-Wallis H test.

### 3.5 Ordered multinomial logistic regression of potential nutritional literacy profiles among the elderly patients with chronic diseases

The three profile categories of elderly chronic disease patients were used as dependent variables: passive dependent type (class 1), cognitive fluctuation type (class 2), and autonomous management type (class 3). Statistically significant indicators from the univariate analysis were used as independent variables in the ordered logistic regression analysis. The assignment of values to the independent variables is shown in Table 4.

**Table 4:** Assignment of independent variables.

Independent variables	Assignment of independent variables
Age	1 = 60–64 years, 2 = 65–74 years, 3 = 75–89 years, 4 = 90 years and above
Monthly personal income (RMB)	1 = ≤1,000 yuan, 2 = 1,001–3,000 yuan, and 3 = 3,001–5,000 yuan, 4 = ≥5,001 yuan
Use of smartphone	1 = yes, 2 = no
Chronic Disease t Self-Efficacy Scale (CDSES)	Raw score

Before conducting the regression analysis, we performed a multicollinearity test on all independent variables. The variance inflation factor (VIF) w-values were all less than five, indicating that there was no serious multicollinearity issues according to commonly accepted thresholds. The test of parallel line test (*χ^2^* = 35.638, *p* = 0.099) showed that the ordered logistic regression model was appropriable. The Pearson goodness-of-fit test (*χ^2^* = 501.239, *p* > 0.05) demonstrated that the model fit the data well. Furthermore, and the likelihood ratio test indicated that the regression model was statistically significant (*p* < 0.001). The ordered logistic regression analysis revealed significant demographic and behavioral determinants of nutritional literacy. Younger patients demonstrated substantially greater odds of better nutritional knowledge retention, showing a clear inverse relationship with advancing age. Income disparities exerted notable effects, with progressively lower monthly earnings corresponding to diminished nutritional literacy levels relative to the highest income bracket. Technology adoption emerged as a positive predictor, with smartphone users exhibiting nearly doubled odds of better nutritional literacy. Furthermore, the analysis identified a dose–response relationship between self-efficacy in chronic disease management and nutritional literacy, with each incremental score improvement conferring proportionally higher odds. These patterns collectively indicate that nutritional literacy in elderly chronic disease patients is dynamically influenced by age-related decline, socioeconomic constraints, technological engagement, and self-management capabilities, as detailed in Table 5.

**Table 5:** Ordered multinomial logistic regression of potential nutritional literacy profiles among the elderly patients with chronic diseases.

Item	*β*	SE	Wald χ^2^	*p*	OR	95% CI
Total chronic disease self-efficacy score	0.034	0.016	4.28	0.039	1.04	1.01–1.07
Age (reference: ≥90 years old)						
60–64 years	3.244	0.827	15.39	0.000	25.64	5.07–129.66
65–74 years	2.634	0.766	11.81	0.001	13.93	3.10–62.61
75–89 years	2.268	0.706	10.31	0.001	9.66	2.42–38.54
Monthly personal income (reference: ≥5,001)						
≤1,000	−2.029	0.631	10.34	0.001	0.13	0.04–0.45
1,001–3,000	−1.171	0.548	4.56	0.033	0.31	0.11–0.91
3,001–5,000	−1.403	0.490	8.20	0.004	0.25	0.09–0.64
Use of smartphone (refer: no)						
Yes	0.672	0.330	4.161	0.04	1.96	1.03–3.74

## 4 Discussion

### 4.1 Nutritional literacy among elderly patients with chronic diseases is in the lower middle range

The results of this study showed that the nutritional literacy score of elderly patients with chronic diseases was 67.50 (51.00, 84.00), which was at a moderate to low level. This finding is consistent with international studies showing that older adults with chronic conditions generally exhibit suboptimal nutritional literacy levels (26). But this result is lower than that of Liu et al. (27) survey on nutritional literacy among elderly individuals (76.28 ± 17.04), which may be related to the following three factors. First, compared with healthy elderly people, elderly patients with chronic illnesses have decreased cognitive ability and social participation due to impaired physical function. This limits their dietary choices and nutritional intake, leading to lower nutritional literacy levels. This cognitive-physical nexus represents a universal challenge observed across different cultural contexts (26). Moreover, the widespread lack of nutritional knowledge and understanding of healthy eating among the elderly population may also be an important reason for their low nutritional literacy. Many elderly patients have insufficient knowledge of nutrients, making it difficult for them to make dietary decisions that meet their health needs. In addition, elderly patients with chronic diseases often rely on family members or social media when obtaining nutrition-related information, a pattern particularly prominent in Chinese cultural context where familial support plays a crucial role in health information acquisition compared to Western counterparts who rely more on healthcare professionals (28, 29). which may result in insufficient accuracy and relevance of the information. Finally, socioeconomic factors also play a significant role. Older chronically ill patients with poorer economic status may face difficulties in access to nutritious food, contributing to their poor performance in nutritional literacy. This economic barrier is universally observed but manifests distinctly in China’s rural–urban divide, where rural elderly demonstrate significantly lower nutritional literacy than their urban counterparts (28). Therefore, improving the nutritional literacy levels of elderly patients with chronic diseases requires a multifaceted approach, including education, social support, and policy interventions. These interventions should incorporate both evidence-based universal strategies and culturally adapted approaches specific to the Chinese context, particularly addressing the unique information-seeking behaviors and rural–urban disparities identified in this population. Such efforts can help them better manage their health conditions.

### 4.2 Different population characteristics of elderly chronic disease patients in different potential categories

In this study, three different nutritional literacy categories of elderly patients with chronic diseases were identified, suggesting that there is heterogeneity in nutritional literacy among elderly patients with chronic diseases. Nutritional literacy is often influenced by demographic factors such as age, education level, economic status, and living environment (30, 31). Globally, these factors consistently shape nutritional literacy profiles, yet their manifestation varies across cultural contexts (26). The results of this study showed that factors such as place of residence, marital status, education level, number of children, type of caregiver, nature of work, type of health insurance, oral health status, and reading habits all showed significant differences (*p* < 0.05) across nutritional literacy categories. For example, “autonomous management type” elderly patients with chronic diseases tended to have higher levels of education and family social support, aligning with universal patterns observed in high-income countries (26) but uniquely mediated in China by self-efficacy as shown in rural populations (28). This may be due to the fact that elderly patients with chronic diseases who have higher education possess greater learning and understanding abilities than those with lower education. Additionally, unlike Western models where health professionals dominate information dissemination, elderly patients in China acquire nutritional knowledge primarily from family members and friends; therefore, higher family social support is associated with higher nutritional literacy levels in this cultural context (29). In addition, differences in living environments may also lead to differences in nutritional literacy among elderly patients with chronic diseases. While urban–rural disparities are universal (26), older adults in Chinese rural areas face compounded challenges due to higher chronic disease comorbidities and economic barriers that exacerbate nutritional inequities (28), further reflecting the impact of urban–rural differences on the health management of older adults (32). For this reason, it is particularly important to develop personalized health education and interventions for older people with chronic diseases in different nutritional literacy categories to ensure accuracy and effectiveness. At the same time, no significant gender differences were found among the nutritional literacy categories of elderly patients with chronic diseases in this study, contrasting with Japanese findings (30). This absence of gender differentiation may reflect methodological and cultural factors specific to the Chinese context. First, the nutritional literacy scale employed in this study may not capture gender-specific dimensions of nutritional knowledge acquisition, as it emphasizes general nutritional knowledge rather than gender-differentiated dietary practices. Second, China’s unique familial structure in elder care, where traditional gender roles become less distinct in later life, may contribute to this finding. As both elderly men and women in multi-generational households often share food preparation and dietary decision-making responsibilities, this creates a homogenizing effect on nutritional literacy (29). Additionally, China’s historical societal transformations, including increased female workforce participation and evolving gender norms over the past decades, may have reduced gender-based disparities in health literacy among current elderly cohorts. Methodologically, potential response biases in self-reported data among elderly Chinese males, who may underreport health knowledge limitations, could also contribute to the observed absence of significant differences. Therefore, nutritional literacy interventions should integrate evidence-based universal frameworks while adapting to China-specific sociocultural dynamics, with future research employing gender-sensitive measurement approaches to better capture potential nuanced differences that may be obscured by current assessment tools.

### 4.3 Age, monthly personal income, use of smartphones, and self-efficacy in chronic disease management are contributing factors to nutritional literacy in elderly patients with chronic diseases

#### 4.3.1 Age

Logistic regression analyses showed that younger elderly chronic disease patients had significantly higher odds of being classified as “autonomous management type” compared to patients aged ≥90 years, as shown in Table 5. However, it is important to exercise caution in interpreting these results, as the odds ratios for age displayed extremely wide confidence intervals, suggesting instability in the estimates and potential issues such as sampling biases or limitations in statistical power. This warrants a critical note that the findings may be influenced by sample characteristics or reduced precision, and clinical conclusions should be drawn tentatively pending further validation. Despite these limitations, this finding is consistent with Patel et al. (31), suggesting that younger older adults with chronic diseases have better nutritional literacy than their older counterparts, which may be attributed to several factors. First, younger older adults generally have higher self-management ability and self-efficacy, both of which positively contribute to their nutritional literacy. Studies have shown that younger older adults are more receptive to new information and technology compared to their senior counterparts, enabling them to acquire and apply nutrition-related knowledge more effectively in managing chronic diseases (33). Second, younger elderly patients tend to be in better physical health and have fewer comorbidities, allowing them to focus more on healthy eating and lifestyle changes. According to Kastner et al., improved health status is closely related to self-management ability, which in turn is directly linked to patients’ level of nutritional literacy (34). In summary, the higher nutritional literacy observed in younger elderly patients with chronic diseases results from a combination of factors, including relatively good health status, stronger learning ability, and higher self-efficacy, providing a solid foundation for improving nutritional literacy in this population. Therefore, in clinical practice, to enhance the nutritional literacy of elderly patients with chronic diseases, medical personnel should develop personalized health management plans aimed at improving physical fitness and self-management ability. Such plans may include Traditional Chinese Medicine (TCM) health exercises like the “Eight Duan Brocade” and encouraging regular physical activity. Additionally, group education programs and individual counseling should be conducted to improve patients’ nutritional knowledge and promote their engagement in self-health management. Through these comprehensive interventions, the overall nutritional literacy level of elderly patients with chronic diseases is expected to improve significantly.

#### 4.3.2 Monthly personal income

Logistic regression analysis showed that the higher the Monthly Personal Income level, the greater the likelihood that elderly chronic disease patients manage their condition independently, as shown in Table 5. This indicates a positive correlation between monthly personal income level and nutrition literacy among elderly chronic disease patients, which aligns with the findings of Gibbs et al. (35) on the nutrition literacy of parents. However, this relationship is not merely economic but is mediated through cognitive-behavioral pathways. As demonstrated in rural Chinese populations, self-efficacy significantly moderates the impact of socioeconomic factors on nutritional literacy (28). The reasons for this were analyzed as follows: firstly, higher income levels are usually associated with better education and health literacy. Studies have shown that patients who are financially well off are more likely to participate in health education and self-management programs, which contribute to their nutrition literacy (36, 37). Notably, these programs’ effectiveness is enhanced when they incorporate digital literacy components, particularly for urban residents with better technology access (29). Secondly, adequate financial resources enable elderly patients to have access to more medical services and health support, including regular health check-ups, chronic disease management courses, and psychological support. Access to these services not only enhances patients’ self-confidence but also promotes their active participation in health management. This creates a syndemic trajectory where financial resources amplify cognitive resources like self-efficacy, creating compounded advantages for higher-income elderly patients (26, 28). In contrast, low-income patients often face financial pressures that limit their investment in health management, which in turn affects their ability to independently acquire nutritional knowledge (38, 39). This is particularly acute in rural areas where limited digital infrastructure further constrains access to online health information, creating a double disadvantage of economic and cognitive barriers (28, 29). In addition, high-income patients usually have a wider range of social support, including support from family members, friends, and community resources, which can provide necessary help and encouragement in the process of patients’ self-management, promote their independent learning of nutrition-related knowledge, and improve their level of nutritional literacy. This support system often includes digital technology assistance from family members, bridging the digital literacy gap for elderly patients (29).

In summary, an increase in the level of Monthly Personal Income not only directly affects the nutritional management ability of elderly chronic disease patients but also indirectly enhances the likelihood of their independent participation in nutritional management and improvement of their nutritional literacy by affecting their education level, ability to access healthcare services, and the accessibility of social support. Therefore, future research should further explore how to improve the ability to manage health of low-income elderly chronic disease patients through policy interventions to enhance their quality of life. Such interventions should address both structural and cognitive dimensions simultaneously—for instance, digital literacy training integrated with economic support programs, particularly targeting rural elderly with chronic conditions (28, 29). For example, the government can increase investment in health education for the elderly and conduct online and offline clinics for nutritional counseling for elderly chronic disease patients, with special attention to developing digital interfaces accessible to low-literacy populations, to help them better understand the management of chronic diseases and the need for self-care. Second, policymakers can reduce the financial burden of low-income elderly patients by providing financial subsidies and access to healthcare services while simultaneously implementing self-efficacy enhancement programs through community health workers to help elderly patients with chronic diseases receive necessary treatments and nutritional support, thus improving their ability to manage their health. In summary, through these integrated policy interventions that address both structural vulnerabilities and cognitive-behavioral processes, not only can the nutritional literacy level of low-income elderly patients be directly improved, but the quality of life and health outcomes of this population can also be enhanced at a broader societal level.

#### 4.3.3 Use of smartphones

Logistic regression analyses showed that older chronic disease patients who used smartphones were more likely to be classified as the “autonomous management type.” This finding is consistent with the results of Gibbs et al. (40), though it should be interpreted with the nuance that this association likely reflects the pattern of usage rather than mere ownership or passive use, requiring further differentiation in health-specific engagement levels. This may be because smartphones specifically utilized for health management purposes—including obtaining nutritional advice, tracking diets, managing medications, and communicating with healthcare providers through dedicated apps—facilitate improved access to nutritional information and resources. These targeted functions enable more effective self-management behaviors that enhance nutritional literacy (41), but future research should dissect specific usage patterns such as frequency, duration, and application types to avoid overgeneralizing the impact of smartphone technology on health outcomes. In summary, smartphones not only assist elderly patients with chronic diseases in obtaining necessary health information but also promote effective self-management in daily life. Medical staff should leverage this trend and actively encourage elderly patients with chronic diseases to use smartphones and related applications to improve their self-management skills and health literacy. Meanwhile, hospitals should actively establish public platforms focused on nutrition for the elderly, especially those with chronic diseases, and carry out nutritional popularization campaigns to enhance nutritional literacy in this population. Furthermore, medical staff should pay attention to elderly chronic disease patients who do not use smartphones. Besides encouraging them to learn how to use smart devices, it is also necessary to provide nutritional knowledge training through offline methods, such as organizing regular nutritional education activities in communities and nursing homes. These efforts aim to improve the coverage of nutritional education, enhance the nutritional literacy of this group, and ultimately improve their clinical outcomes.

#### 4.3.4 Self-efficacy in chronic disease management

Self-efficacy in illness refers to an individual’s confidence and belief in their ability to manage and cope with illness. Research indicates that patients with higher self-efficacy can overcome specific fears, demonstrate greater engagement in treatment and adherence, and tend to have better health outcomes after disease treatment (42, 43). However, the manifestation of self-efficacy is not uniform but interacts significantly with structural variables such as income and residence location, creating syndemic trajectories that shape health outcomes (26). In this study, the disease self-efficacy score of elderly chronic disease patients was 47.00 (38.00, 52.00), with the symptom management self-efficacy score at 31.00 (25.00, 34.75) and the co-management self-efficacy score at 16.00 (13.00, 18.00), reflecting a moderate to high level. This finding is consistent with the results of Chen et al. (44), further supporting evidence of elderly patients’ self-management abilities. These results indicate that elderly patients with chronic diseases generally have a high level of disease self-efficacy. Yet when analyzed through syndemic frameworks, we observe that high self-efficacy alone is insufficient to overcome structural barriers—low-income rural patients with high self-efficacy still showed 30% lower nutritional literacy than urban high-income peers with equivalent self-efficacy scores (28). Therefore, clinical staff should actively engage and support patients in utilizing their self-management abilities when implementing interventions, with particular attention to contextualizing support based on residential and economic circumstances, with the goal of enhancing patients’ disease management skills and ultimately improving their health outcomes.

Logistic regression analyses in this study showed that older patients with chronic diseases who had a higher level of disease self-efficacy had a higher probability of being classified as “autonomous management type” (*β* = 0.034, OR = 1.04, *p* = 0.039). This indicates that disease self-efficacy promotes better nutritional status among older patients with chronic diseases, which aligns with Zhang (45) findings on nutritional health behaviors for the elderly. Possible reasons for this include that patients with high self-efficacy usually have stronger self-monitoring and problem-solving abilities, enabling them to adopt positive coping strategies when facing health challenges, thus improving their overall health status. Critically, these abilities are amplified when supported by digital health tools and economic resources, creating compounded advantages for structurally advantaged groups (26). In addition, increased self-efficacy may encourage healthier choices in nutritional intake and enhance patients’ knowledge and practice of healthy eating (27). Studies have shown that patients with high self-efficacy are more inclined to adopt effective self-management strategies to improve their overall health. This not only helps to enhance their perception and practice of healthy eating but also establishes positive psychological coping mechanisms (46).

Therefore, disease self-efficacy plays a key role in the nutritional literacy and health management of elderly patients with chronic diseases, and clinical staff should further explore ways to enhance patients’ self-efficacy through educational and supportive measures that simultaneously address structural barriers aimed at improving their health outcomes. To achieve this goal, nutrition management strategies for older patients with chronic diseases should include personalized self-management plans. For example, health education, nutritional counseling, and psychological support can be provided through digitally adapted platforms for rural patients and income-supplement programs for low-income groups to help patients set reasonable health goals and enhance their self-monitoring and problem-solving abilities (47). These interventions can not only improve patients’ self-efficacy but also promote more positive coping strategies in the face of health challenges, particularly when integrated with structural support systems, thereby further improving their quality of life and health outcomes.

## 5 Shortcomings and prospects

This study has the following shortcomings due to limitations in manpower and time: (1) As a single-center urban tertiary hospital-based investigation with a sample size of 316 elderly chronic disease patients, the generalizability of findings is constrained by geographic and cultural homogeneity. The convenience sampling approach may overrepresent higher socioeconomic groups, potentially biasing latent class distributions toward profiles more common in urban settings; (2) The cross-sectional design limits causal inferences about nutritional literacy trajectories. While latent class analysis identified distinct profiles, longitudinal validation is needed to evaluate class stability and health outcome associations; (3) Smartphone utilization was documented as an influencing factor, but granular analysis of usage patterns (e.g., frequency, duration, application types) and their specific roles in nutrition education delivery remains unexplored; (4) The identified correlation between chronic disease self-efficacy and nutritional literacy warrants expanded investigation into mediating mechanisms and behavioral pathways to strengthen clinical applicability. However, it should be particularly noted that this study only conducted a cross-sectional investigation, which cannot explain the dynamic influence mechanism between chronic disease self-efficacy and the level of nutrition literacy. Additionally, due to the potential underestimation of regional and cultural differences in the determinants of nutrition literacy, the generalizability of the research results may be limited.

To overcome these limitations, future research should consider the following directions. First, expanding the sample size and conducting a multi-center survey to ensure that the findings more comprehensively reflect the level of nutritional literacy of elderly patients with chronic diseases in different regions and cultural contexts. Future multi-center studies incorporating stratified rural sampling and prospective designs are recommended to enhance external validity. This can be achieved by collaborating with multiple healthcare organizations to ensure a diverse and representative sample. Second, future studies should adopt a longitudinal design to track changes in nutritional literacy and its impact on health outcomes over time. By regularly assessing the level of nutritional literacy in the same group of elderly patients, its evolution over time and its causal relationship with health outcomes can be better understood. In addition, more refined quantitative tools could be considered to assess the dimensions of nutritional literacy. Third, regarding smartphone use, future studies should delve into specific usage patterns and their application in nutrition education. Specialized questionnaires could be designed to assess the frequency, modes, and effects of using smartphones to access nutritional information, and to explore how smartphone apps can be used to improve nutritional literacy in this population. Finally, future studies could explore more relevant variables, such as social support and mental health status, to analyze in depth the relationship between chronic disease management self-efficacy and nutritional literacy. This will help to build a more comprehensive theoretical framework for understanding nutritional literacy and its impact on health management in elderly chronic-disease patients. Through these measures, future research will be able to provide a more robust scientific basis for improving nutritional literacy and health outcomes in older patients with chronic diseases.

## 6 Conclusion

In summary, this study found that the nutritional literacy of elderly patients with chronic diseases was moderately low, indicating a widespread need for nutrition education within this population. A lack of nutritional literacy can lead to inadequate knowledge of health status, which negatively impacts chronic disease management and treatment outcomes. The relationship between nutritional literacy and health outcomes is especially critical among elderly patients with chronic diseases. By applying latent profile analysis (LPA), we identified three distinct categories of nutritional literacy, demonstrating significant individual differences among these patients. Moreover, patients in different categories exhibited significant differences in socioeconomic factors, including place of residence (urban vs. rural), marital status (married vs. unmarried), and education level (high school and above vs. junior high school and below). These differences provide a foundation for developing individualized nutritional interventions. Notably, patients classified as “passive dependent type”—those who rely heavily on others for nutritional information and support may require additional social support and educational resources to enhance their nutritional literacy.

In addition, age, Monthly Personal Income, Use of Smartphone, and Self-efficacy in chronic disease management were shown to be important contributors to nutritional literacy among older patients with chronic diseases. These findings emphasize that in the management of chronic diseases in the elderly, in addition to focusing on the disease itself, attention should be paid to patients’ nutritional education and skill training to improve their self-management abilities and promote better health outcomes. Therefore, clinical practitioners should carry out nutritional education interventions for elderly chronic disease patients, such as providing personalized nutritional guidance, conducting community nutritional talks, and promoting peer support groups among patients. These initiatives can enhance patients’ knowledge of their own health. They can also boost patients’ motivation and self-confidence in participating in chronic disease management, thereby improving treatment outcomes. In addition, policy makers should consider incorporating nutrition education into the standard process of chronic disease management for the elderly to ensure that all elderly patients have access to the necessary nutritional support. This will not only help improve patients’ quality of life, but also reduce the burden on the healthcare system. Further research should first focus on the specific needs of patients in different nutritional literacy categories, and explore education and intervention strategies that suit their characteristics. For example, for “passive dependent type” patients—those who rely heavily on others for managing their nutrition—consideration can be given to introducing family members or community volunteers to participate in their nutrition education to enhance the patients’ motivation and practical abilities, such as following dietary advice and preparing nutritious meals. At the same time, the use of modern technology, such as mobile phone applications, to provide regular nutritional information and online counseling services can complement these approaches and help improve patients’ nutritional literacy. In conclusion, improving the nutritional literacy of elderly patients with chronic diseases is an important part of enhancing health outcomes and requires multifaceted efforts and sustained attention. Through comprehensive nutrition education and personalized intervention strategies, we expect to significantly improve the health status and quality of life of these patients.

## Data Availability

The original contributions presented in the study are included in the article/supplementary material, further inquiries can be directed to the corresponding author.
